# Alginate–Poly[2-(methacryloyloxy)ethyl]trimethylammonium Chloride (PMETAC) Immunoisolating Capsules Prolong the Viability of Pancreatic Islets In Vivo

**DOI:** 10.3390/biomedicines12112573

**Published:** 2024-11-10

**Authors:** Polina Ermakova, Ekaterina Vasilchikova, Arseniy Potapov, Maxim Baten’kin, Liya Lugovaya, Alexandra Bogomolova, Julia Tselousova, Alexey Konev, Natalia Anisimova, Alena Egoshina, Mariya Zakharina, Nasipbek Naraliev, Denis Kuchin, Vladimir Zagainov, Sergey Chesnokov, Aleksandra Kashina, Elena Zagaynova

**Affiliations:** 1 Federal State Budgetary Institution of Higher Education “Privolzhsky Research Medical University” of the Ministry of Health of Russia, 603005 Nizhny Novgorod, Russia; vasilchikova_ea@pimunn.net (E.V.); potapov_al@pimunn.net (A.P.); liya.lugovaya@inbox.ru (L.L.); bogomolova_a@pimunn.net (A.B.); julia@yandex.ru (J.T.); nasip_95_kg@mail.ru (N.N.); pomc.kuchin@gmail.com (D.K.); zagainov@gmail.com (V.Z.); meleshina_a@pimunn.net (A.K.); ezagaynova@gmail.com (E.Z.); 2 Federal State Educational Institution of Higher Educational Institution “National Research Nizhny Novgorod State University Named After N.I. Lobachevsky”, 603022 Nizhny Novgorod, Russia; 3 Federal State Budgetary Institution of Science Institute of Organometallic Chemistry Them G.A. Razuvaev Russian Academy of Sciences, 603950 Nizhny Novgorod, Russia; batenkinmax@iomc.ras.ru (M.B.); alex-kon@mail.ru (A.K.); nata.d.anisimova@yandex.ru (N.A.); lokteva@iomc.ras.ru (A.E.); m.zakharina@mail.ru (M.Z.); sch@iomc.ras.ru (S.C.); 4 Nizhny Novgorod Regional Clinical Hospital Named After N.A. Semashko, 603126 Nizhny Novgorod, Russia; 5 State Budgetary Healthcare Institution “Nizhny Novgorod Regional Clinical Oncology Dispensary”, 603163 Nizhny Novgorod, Russia; 6 Federal Scientific and Clinical Center for Physico-Chemical Medicine Named After Academician Yu. M. Lopukhin, 119435 Moscow, Russia

**Keywords:** microcapsules, microencapsulation, alginate, islet of Langerhans

## Abstract

Background/Objectives: This study focuses on the development and evaluation of novel alginate–poly[2-(methacryloyloxy)ethyl]trimethylammonium chloride (PMETAC) microcapsules for encapsulating pancreatic islets to address insulin deficiency in diabetes. Methods: In previous research, we fabricated and characterized PMETAC microcapsules, evaluating their stability and permeability in vitro. This study further probes the capsules in vivo, focusing on the functional activity of the encapsulated islets post-transplantation, their viability extension, and the assessment of the immunoprotective, antifibrotic properties, and biostability of the capsules. Results: Rabbit-derived islets were encapsulated and transplanted into diabetic rats. The encapsulated islets maintained insulin secretion for up to 90 days, significantly longer than non-encapsulated ones, which ceased functioning after 7 days. Histological analysis demonstrated high biocompatibility of the PMETAC coating, resulting in minimal fibrotic overgrowth around the capsules. Conclusions: The study highlights the critical role of immunoprotection and the tendency to reduce fibrosis in prolonging islet function. These findings suggest that PMETAC-coated capsules offer a promising solution for cell-based therapies in diabetes by improving graft longevity and reducing fibrotic overgrowth.

## 1. Introduction

Type 1 diabetes is a chronic disease characterized by impaired insulin production, leading to elevated blood glucose levels. Hyperglycemia poses significant risks and can cause severe complications such as cardiovascular diseases and kidney failure [1]. This condition affects millions of people worldwide, with a prevalence rate of 9.5% [2]. The primary treatment method is insulin therapy; however, even modern approaches fail to provide complete protection against glycemic events and chronic complications [3]. A promising alternative is the transplantation of insulin-producing cells, which may offer more stable glucose control and reduce the risk of complications, as biological cells are more reliable than artificial systems [4].

Insulin-producing cells can be sourced from pancreatic islets or generated from stem cells. However, immune rejection and therefore the need for immunosuppressive therapy remain significant challenges to successful transplantation. Chronic immunosuppression is associated with side effects, such as kidney dysfunction, increased susceptibility to infections, and a higher risk of cancer. Moreover, immunosuppressive drugs can adversely affect the transplanted cells themselves, increasing the likelihood of graft failure [5]. To address these issues, alternative approaches are being developed, including the induction of immune tolerance, the creation of immunoprivileged β-cells, and the use of encapsulation [6].

Cell encapsulation is a promising alternative to traditional immunosuppression, creating a physical barrier between the immune system and the graft. This capsule allows oxygen, insulin, and nutrients to reach the cells while preventing immune attacks [7]. Additionally, encapsulation enhances the survival and functionality of the insulin-producing cells post-transplantation [5,8]. Encapsulation technology is being actively developed by academic and industrial research centers worldwide [9]. Among various polymers, alginate is considered the most suitable material for drug delivery and cell therapy due to its biocompatibility, rapid gel formation under physiological conditions, biodegradability, non-immunogenicity, and the possibility of polymer modification [8]. There are currently diverse encapsulation approaches (nano-, micro-, and macroencapsulation) utilizing different technologies such as microfluidics, 3D printing, the photo- and thermal cross-linking of polymers, or surfactant-activated in situ gelation. Such encapsulation technologies can be further improved by advancing capsule polymer materials or co-encapsulating cells with peptides, drugs, and other agents [8].

Nevertheless, several challenges continue to hinder the effectiveness of encapsulated cell transplantation. These include fibrotic overgrowth around the capsules, leading to nutrient deprivation and hypoxia impacting on the cells [10]; impaired vascularization, which also causes hypoxia; loss of capsule biostability, compromising its integrity; and a lack of capsule biocompatibility, which is essential for clinical applications [8]. All these factors limit the long-term functionality and survival of encapsulated cells [11]. Therefore, improving encapsulation technologies and developing new, biocompatible materials is critical for enhancing cell survival after transplantation [5].

The polymer poly-[2-(methacryloyloxy)ethyl]trimethylammonium chloride (PMETAC) shows promise as a material for creating new microcapsules. It is a structural analog of poly-L-lysine and has demonstrated good biocompatibility. The surface modification of poly(ether ketone) substrates using PMETAC has been shown to significantly reduce fibrinogen adsorption and implant overgrowth [12]. Based on our previous research, capsules composed of a new combination of alginate and PMETAC have demonstrated stability and selective permeability in vitro. This study aims to investigate the properties of novel capsules designed to enhance the correction of insulin deficiency using pancreatic islets. Key aspects include preserving the functional activity of the encapsulated islets post-transplantation and the ability of the capsules to extend the duration of this activity. Additionally, there is focus on the immunoprotective and antifibrotic properties of the capsules, as well as their biostability. These characteristics are crucial for ensuring the effective and safe application of capsules in the treatment of diabetes and other glucose regulation disorders.

## 2. Materials and Methods

### 2.1. Animals

The study used two-month-old rabbits, weighing 2–3 kg, as pancreas donors for islet isolation, and 2–3-month-old rats, weighing 200–300 g, for testing streptozotocin (STZ)-induced diabetes and for conducting transplantation experiments with encapsulated islets. All our studies with experimental animals were approved by the local ethics committee of the Privolzhsky Research Medical University (protocol № 10; date: 26 June 2020).

### 2.2. Materials for Capsule Synthesis

Low-viscosity sodium alginate («Aldrich», A1112, Saint Louis, MI, USA), dextran with an average molecular weight of 20,000 Da (A0 «Vector», Novosibirsk, Russia), polyethylene glycol with an average molecular weight of 8000 Da (PEG-8000, PAO “Nizhnekamskneftekhim,” Russia), barium chloride dihydrate (Khimreaktiv, Russia), [2-(methacryloyloxy)ethyl]trimethylammonium chloride Aldrich, (408107, Saint Louis, MI, USA), tris (hydroxymethyl) aminomethane (Aldrich, Saint Louis, MI, USA), and methylene blue (Khimreaktiv, Russia) were used without further purification. Poly[2-(methacryloyloxy)ethyl] trimethylammonium chloride was synthesized [13] by the polymerization of the [2-(methacryloyloxy)ethyl]trimethylammonium chloride according to the method described in Lin et al. [14].

### 2.3. Characterization of the PMETAC Polymer

The IR spectrum of the synthesized polymer was recorded using an FT-801 IR Fourier spectrometer with an ATR attachment (OOO NPR Simeks, Novosibirsk, Russia). The polymer was placed on a diamond substrate for the ATR attachment and pressed using the built-in press.

The determination of the molecular weight distribution of the polymer samples was carried out by gel permeation chromatography using a high-performance liquid chromatograph, the LC-20AD (Shimadzu, Japan). Analysis conditions: eluent 0.5 N acetic acid solution, flow rate 0.8 mL/min, T = 30 °C, ELSD detector (low temperature evaporative light scattering detector). Column: TSK-GEL G3000SWXL, 7.8 mm ID × 30.0 cm L, 5 µm.

### 2.4. Islet Isolation

The source pancreas was digested with a solution of collagenase V (Collagenase from *Clostridium histolyticum*, Sigma, Saint Louis, MI, USA) in a modified Hanks solution containing added CaCl2. Following perfusion, the pancreas was incubated on a preheated shaker at 37 °C for 11–15 min. Throughout the digestion process, the pancreatic tissue gradually softened until the entire organ was broken down into small granules. Digestion was terminated by the addition of Hanks’ Balanced Salts Solutions (HBSS) supplemented with 5% BSA. The purification of the islets of Langerhans from the surrounding exocrine tissue was accomplished by passing the mixture three times through a metal sieve with a 0.5 mm mesh size. Any undigested tissue that remained was discarded. The resulting suspension was centrifuged at 200× *g* for 3 min, after which the supernatant was carefully removed. The resulting pellet was then subjected to density gradient centrifugation using Ficoll DL-400 (1.095, 1.084, 1.072, and 1.048 mg/L) (Sigma, Saint Louis, MI, USA) at 800× *g* for 15 min. After centrifugation, the islets were washed with MHBS. The isolated islets were then cultured in RPMI medium (Gibco, London, UK) with low glucose, supplemented with L-glutamine (0.58 mg/mL) (PanEco, Moscow, Russia), 10% fetal bovine serum (FBS) (Gibco, London, UK), and an antibiotic–antimycotic solution (Antibiotic-Antimycotic 100X, ThermoScientific, Waltham, MA, USA) at 37 °C in a 5% CO_2_ atmosphere. During the isolation procedure, dithizone staining was used to identify the islet cells, and the stained cells were visualized using a Leica DM2500 microscope (Leica, Berlin, Germany).

### 2.5. Capsule Synthesis

Alginate microdroplets containing the pancreatic islets were formed using a microfluidic setup, which utilizes external manipulation of the continuous phase flow [13]. The microfluidic setup is protected by know-how No. 249/Axd dated 9 February 2022. Prior to the experiment, the microfluidic device was cleaned with alcohol and UV-sterilized for 15 min, and all solutions used were aseptically processed. A suspension of islets in a solution of sodium alginate and dextran in phosphate-buffered saline was used as the dispersed phase. To form this, islets in a nutrient medium were added to the alginate/dextran solution at a 1:3 volume ratio immediately before encapsulation. The concentration of islets in the final encapsulation suspension was 15,000 per milliliter. The final mixture contained sodium alginate and dextran at concentrations of 2% and 15% by weight, respectively. A solution of polyethylene glycol (30% by weight) in distilled water was used as the continuous phase.

The microdroplets obtained using the microfluidic method were cross-linked with barium ions to form hydrogel capsules. The concentration of barium chloride in the gelling solution was 2.9% by weight. After this and each subsequent step, the microcapsules were washed in a buffer solution based on tris(hydroxymethyl)aminomethane (0.45% by weight) and physiological saline with a pH of 7.2. Then, a semipermeable polymer membrane was formed on the surface of the microcapsules by incubating them in a PMETAC solution (concentration to be specified) in the washing buffer for 10 min. Next, an additional alginate coating was applied to the surface of the polymer membrane by incubating the microcapsules in a 0.2% by weight sodium alginate solution in physiological saline for 5 min. Finally, the microcapsules containing islets were placed in a nutrient medium for further studies.

### 2.6. Diabetes Modeling

Diabetes was induced following a 12 h fast. The animals were injected with streptozotocin (STZ), freshly dissolved in citrate buffer (pH 4.5), at a dose of 45 mg/kg of body weight, administered intravenously to the rats (*n* = 15). STZ-induced diabetes was considered successfully established if the blood glucose level of the rats exceeded 16 mmol/L after a 7-day induction period. To confirm the formation of a stable STZ-induced diabetes model, blood glucose levels were monitored over a 3-month period and at the end of the experiment. The plasma insulin levels in both healthy rats and those with STZ-induced diabetes were also analyzed. The immunohistochemistry (IHC) of the islets in the pancreas was also determined for insulin and glucagon.

To ensure the stability of the STZ-induced diabetes model at a dose of 45 mg/kg (*n* = 15), insulin therapy was administered to counteract glucose toxicity. Exogenous insulin, Humulin^®^ NPH (Lilly France, France), was injected subcutaneously twice daily.

In preliminary experiments, we had used an STZ dose of 65 mg/kg and optimized the insulin therapy dose. During the first 14 days after diabetes induction, insulin was administered at doses of 1–3 IU per day, and the blood glucose level averaged 21.9 ± 4.9 mmol/L. Due to significant insulin resistance in the rats, the insulin dose was increased to 7–8 IU per day, with an average blood glucose level of 20.6 ± 3.4 mmol/L. By day 25 of therapy, the glucose level had decreased to 10.0 ± 4.7 mmol/L.

Subsequently, the insulin dose was adjusted to 6–7 IU per day, depending on the glycemic levels. For further studies, an STZ dose of 45 mg/kg was selected, as insulin therapy for STZ-induced diabetes at this dose resulted in more stable and controlled blood glucose levels compared to insulin therapy for diabetes induced with an STZ dose of 65 mg/kg.

### 2.7. Experimental Design (Transplantation of Encapsulated and Non-Encapsulated Islets)

To study the properties of the capsules, both encapsulated and non-encapsulated islets were transplanted into the omental tissue of the rats. We selected this transplantation site because it is highly vascularized and secretes various growth factors (e.g., CXCR4, VEGF, and SDF-1) that promote islet vascularization and survival. The omentum provides a sufficiently large space for capsule transplantation and is also suitable for the transplantation of non-encapsulated islets. Transplantation of non-encapsulated islets into the omentum with thrombin has been clinically tested in the U.S. (NCT02213003).

Rabbits were chosen as islet donors because xenogeneic material enhances the immune response, making it more suitable for studying immune reactions to foreign cells. This protocol is also employed by other researchers [15,16,17].

Three experimental groups with STZ-induced diabetes were created: untreated (*n* = 15), encapsulated islet transplantation (*n* = 10), and non-encapsulated islet transplantation (*n* = 10). The untreated animals served as the control group. It is important to note that the blood glucose level of the untreated rats was 28.8 ± 4.0 mmol/L. Therefore, it was decided that when the blood glucose level in rats with transplanted encapsulated or non-encapsulated islets reached 27 mmol/L during on two consecutive measurements, the graft was considered non-functional. For testing, some animals were euthanized immediately after the loss of graft function, while others were euthanized after either 2 weeks, 1 month, or 3 months for histological analysis of the transplanted material. Animals with still-functional islets were also euthanized 3 months post-transplantation.

### 2.8. Transplantation of Encapsulated and Non-Encapsulated Islets

Prior to surgery, the animals were anesthetized with Zoletil (Virbac, Hamilton, New Zealand) at 6 mg/kg and xylazine (Bayer, Leverkusen, Germany) at 90 mg/kg. For the transplantation of encapsulated islets into the omentum, a small incision (0.5–0.7 cm) was made in the peritoneal region. The omental tissue was exposed and spread on moist gauze. A total of 2000 encapsulated or non-encapsulated islets in 1 mL of salineBaye were transplanted into the omentum using a G18 needle. The omentum was then placed back into the abdominal cavity, and the incision was sutured [18]. Following transplantation, the rats were treated with antibiotics for 3–5 days (Augmentin, 1 mg/mL).

### 2.9. Statistical Analysis

The statistical analyses were conducted using GraphPad Prism 9.0 software (GraphPad Software, La Jolla, CA, USA). Data were expressed as the median ± quartiles 25–75. Non-parametric data were analyzed using the Mann–Whitney U test. A *p*-value of less than 0.05 was considered statistically significant.

## 3. Results

### 3.1. Capsule Synthesis

For the in vivo studies, alginate microcapsules containing Langerhans islets were formed using a laboratory experimental setup [13]. The microdroplets made of alginate composite, generated by the microfluidic method, were cross-linked with barium ions to form hydrogel capsules. To enhance stability, biocompatibility, and the functional activity of the encapsulated islets, a semipermeable polymer membrane made of PMETAC was formed on the surface of the microcapsules.

### 3.2. Confirmation of the Stability of Streptozotocin-Induced Diabetes

To further assess the properties of the capsules, the stability of STZ-induced diabetes in the rats was confirmed after the removal of glucotoxicity. Existing evidence suggests the potential for β-cell regeneration upon the alleviation of glucotoxicity, at least over long periods (120 days) [19,20]. Therefore, we decided to verify the stability of the model over a 90-day period.

**Figure 1:**
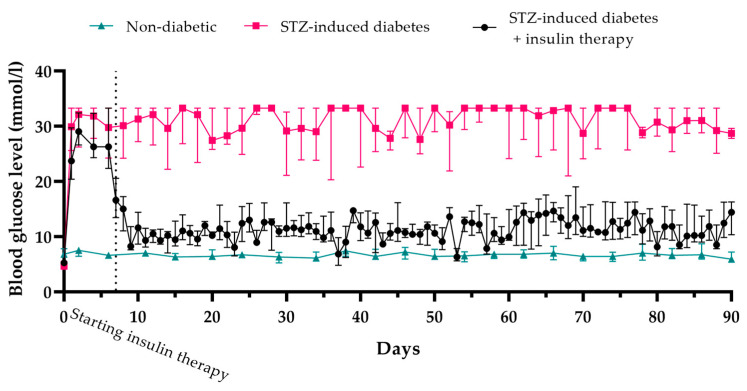
Blood glucose levels in STZ-induced diabetic rats without treatment and with insulin therapy (median ± quartiles 25–75). All three experimental groups were statistically significantly different from each other (*p* < 0.05).

The mean blood glucose level in healthy animals was 6.7 ± 0.3 mM, while in rats with STZ-induced diabetes, it rose to, and remained at, 28.8 ± 4.0 mM over the three-month duration of the experiment. The insulin level in the STZ-induced diabetic rats was 1.8 µU/mL, compared to 17.1 µU/mL in healthy rats (Figure 1).

To alleviate glucotoxicity, insulin therapy was administered at doses of 6–8 IU per day (2–3 IU in the morning and 4–5 IU in the evening). Throughout the therapy, blood glucose levels remained at 11.2 ± 3.7 mM.

**Figure 2:**
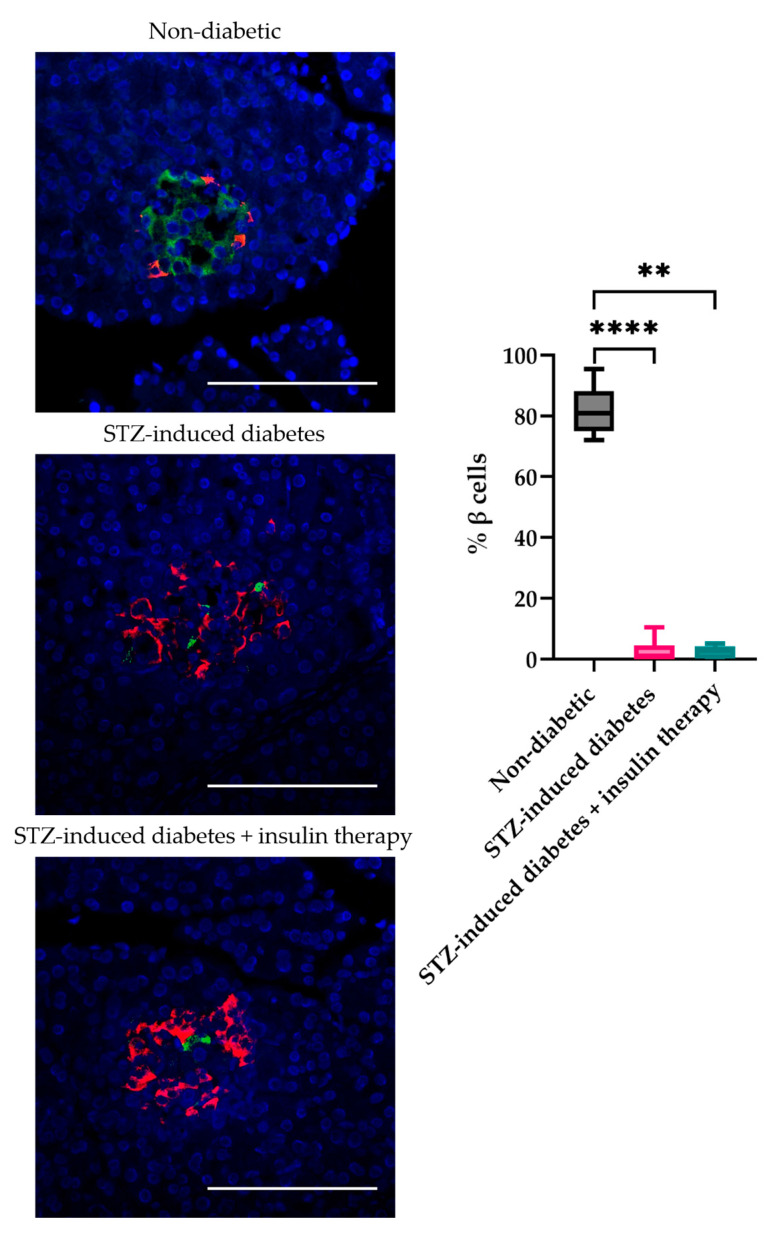
Analysis of the number of β-cells in the islets of the pancreas of rats with untreated diabetes and those receiving insulin therapy, 3 months after the induction of diabetes. Fluorescence images showing islets labeled with insulin producing cells (green) and glucagon producing cells (red), cell nuclei (blue). (Scale bar 100 μm). **, **** *p* < 0.01, 0.001, respectively, compared with non-diabetic group.

Immunohistochemical analysis revealed that β-cell regeneration did not occur in STZ-induced diabetic rats, either untreated or with insulin therapy, during the entire experiment (Figure 2). This indicates that the removal of glucotoxicity did not lead to the restoration of pancreatic endocrine function by three months. In normal animals, the number of β-cells per islet was 81.9 ± 7%, while in STZ-induced diabetic animals, it was significantly reduced to 3.1 ± 3%. In diabetic animals undergoing insulin therapy, the number of β-cells was further decreased to 2 ± 1.9%. The reduction in blood glucose levels in STZ-induced diabetic rats was therefore solely attributable to the insulin therapy and not to the recovery of endogenous β-cells.

Thus, the administration of STZ at a dosage of 45 mg/kg induces stable diabetes over a three-month period, both in the untreated condition and following glucotoxicity alleviation.

### 3.3. In Vitro Confirmation of Islet Quality in the Capsules

**Figure 3:**
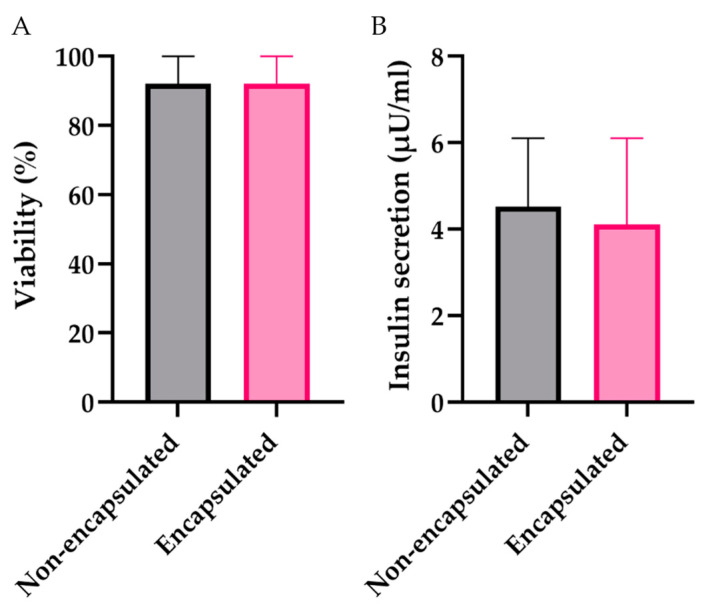
Graph of changes in the (**A**) viability and (**B**) functional activity of pancreatic islets before and after encapsulation. The non-encapsulated islets are marked in gray, while the encapsulated islets are marked in pink. No statistically significant difference was found between the non-encapsulated and encapsulated islets in terms of both viability and functional activity.

**Figure 4:**
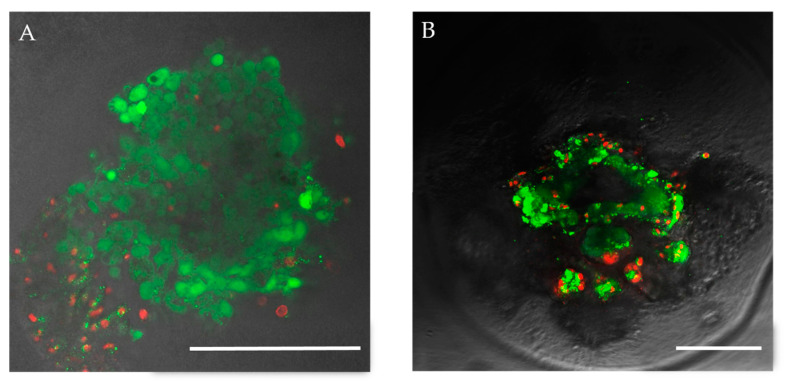
Confocal fluorescence images showing islets (**A**) without and (**B**) with encapsulation, labeled with viability stains Calcein AM (green) and propidium iodide (red) (scale bar 100 μm).

Previous studies have demonstrated that the encapsulation process preserves both the viability and functional activity of the islets [13]. In this study, to confirm the quality of the islets post-encapsulation, we assessed the viability and functional activity of both the encapsulated and non-encapsulated rabbit islets. For further encapsulation, islet isolation from rabbits was performed using a standard protocol with custom modifications [21]. This protocol yields approximately 5000 ± 500 islets per rabbit. After isolation, 95.7 ± 4% of the rabbit islet cells remained viable (Figure 3A and Figure 4). The assessment of islet cell viability showed no statistically significant difference in the number of viable cells after encapsulation (93.1 ± 4%, *p* = 0.07).

To evaluate the functional activity, ELISA was used to measure insulin levels in the culture medium after 24 h of incubation of the islets, both encapsulated and non-encapsulated, with a loading of 300 islets/mL. No statistically significant difference was found between the non-encapsulated and encapsulated islets (4.62 ± 1 μU/mL and 4.25 ± 1 μU/mL, respectively, *p* = 0.59) (Figure 3B). Therefore, it can be concluded that the islets can obtain all necessary nutrients, remain viable, and continue to synthesize insulin both before and after encapsulation, making them suitable for transplantation.

### 3.4. Assessment of the In Vivo Functional Activity of Encapsulated Islets and the Ability of Capsules to Prolong This Activity Compared to Non-Encapsulated Islets

The functional activity of encapsulated and non-encapsulated islets in vivo was evaluated by measuring changes in the blood glucose levels in rats with STZ-induced diabetes after transplantation. The transplant was considered functionally active if the glucose levels in the rats were below 28.8 ± 4.0 mM.

Following transplantation of non-encapsulated islets, the blood glucose levels of the rats decreased to 23.2 ± 8.5 mM, which was statistically significantly lower compared to the untreated STZ-induced diabetic group (*p* = 0.03). Thus, the islet transplantation reduced blood glucose levels in these rats. However, the functional duration of non-encapsulated xenogeneic islets varied, lasting at most 7 days. After this period, the blood glucose levels of all experimental animals returned to over 27 mM (*p* = 0.17), indicating a loss of graft functionality.

**Figure 5:**
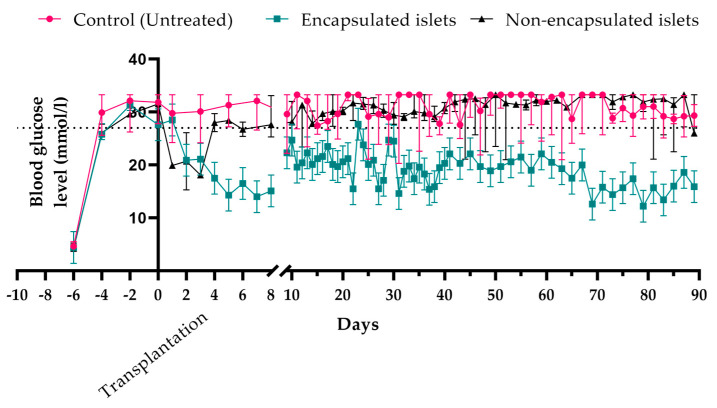
Blood glucose levels in STZ-induced diabetic rats following the transplantation of encapsulated and non-encapsulated islets (median ± quartiles 25–75). Following the transplantation of non-encapsulated islets, blood glucose levels in the rats decreased for 7 days, which was statistically significantly lower compared to the untreated STZ-induced diabetic group (*p* = 0.03). After this period, blood glucose levels in all animals with non-encapsulated islet transplantation were not statistically different from the control (untreated) group (*p* = 0.17). In contrast, the transplantation of encapsulated islets led to a significant reduction in blood glucose levels that persisted for up to 90 days, which was statistically different from the untreated control group (*p* < 0.0001).

**Figure 6:**
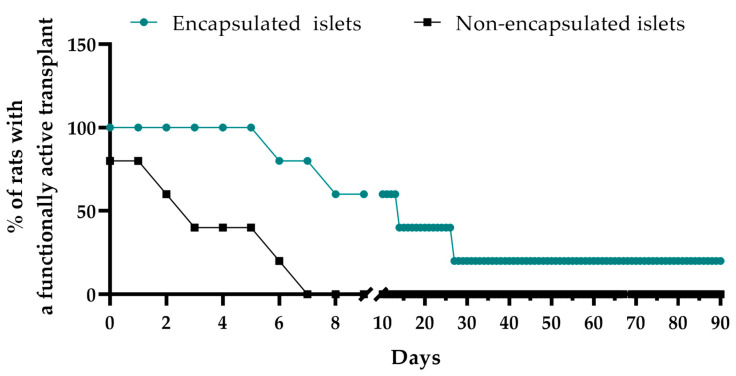
Assessment of the duration of action of the transplant (encapsulated and non-encapsulated islets) in STZ-induced diabetic rats.

The main experimental group of rats received encapsulated islets. Upon transplantation of these islets, the rats’ blood glucose levels initially decreased to 23.5 ± 4.3 mM within the first three days and then remained at 19.3 ± 4.4 mM until day 90 of observation. The blood glucose levels after this time in these treated rats were therefore reduced by 33% (*p* < 0.0001) compared to the untreated animals. This confirmed the preservation of islet functionality within the capsules (Figure 5 and Figure 6).

However, although more than 50% of the rats showed a loss of encapsulated islet functionality 14 days post-transplantation, in 20% of the animals, the graft maintained functional activity until day 30, and this functional activity persisted in 20% of the animals until day 90. These findings suggest that the capsules can prolong islet functionality. Moreover, since xenogeneic islets were used for encapsulation, and these would normally trigger immune system activation, the results also demonstrate the effectiveness of the immunoprotective properties of the capsules.

### 3.5. Anti-Fibrotic Properties and Biocompatibility of Islet Capsules

**Figure 7:**
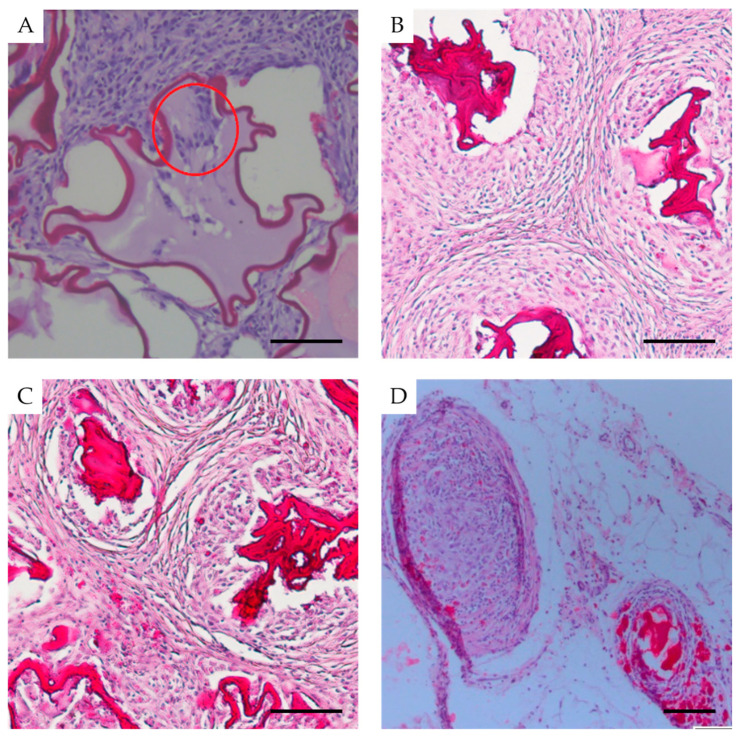
Representative images illustrating the response of surrounding tissues in rats to encapsulated islets transplantation. (**A**) 2 weeks after transplantation (functionally active transplant) islet is highlighted in red. (**B**) 2 weeks after transplantation (NOT functionally active transplant (rat blood glucose level above 27 mmol/L)), (**C**) 1 month after transplantation, and (**D**) 3 months after transplantation. Scale bar 100 µm.

The anti-fibrotic properties and biocompatibility of the capsules were evaluated through a histological analysis of the omental tissues after the loss of graft functionality. However, tissues from the omentum with a functionally active graft were also examined in a single case 14 days post-transplantation (as detailed in Materials and Methods). In this case, 48% of the microcapsules had maintained an intact membrane and a clearly defined cavity, with islets visualized within some of the capsules (Figure 7A). Two-thirds of the microcapsules were surrounded by developing connective tissue with a high cell density and diffuse cell distribution that included macrophages (47%), fibroblasts (28%), lymphocytes (22%), granulocytes (2%), and mast cells (1%). One-third of the microcapsules were surrounded by omental adipose tissue, with isolated cells such as fibroblasts, lymphocytes, granulocytes, and mast cells present between the microcapsules.

In rats where the implanted microcapsules had lost functionality after 14 days, a chronic inflammatory response was observed, with young connective tissue forming around the microcapsules. The inflammatory infiltrate consisted primarily of lymphocytes and macrophages (reactivity ranking 2–3). Giant multinucleated cells were observed near the microcapsules in 15–20% of cases. Additionally, although there were no signs of capsule wall destruction, 20–40% of the microcapsules had lost their integrity, leading to the infiltration of inflammatory cells (neutrophils, lymphocytes) into the capsules, resulting in islet cell death (Figure 7B). The young connective tissue contained a high number of fibroblasts and was composed of relatively thin collagen fibers, with the thickness of the connective tissue layer measuring 44 ± 11 µm. Thus, the rate of biodegradation and loss of graft functionality was determined by the individual responses of the animals.

In rats with microcapsules that lost functionality after one month (Figure 7C), a more pronounced macrophage response was observed. The microcapsule walls were thickened (likely due to swelling) and infiltrated by lymphocytes, with a macrophage layer forming around the capsules. Foreign-body giant cell (FBGCs) were found on the surface of 50–60% of the microcapsules. Most of the microcapsules had lost their original structure, with the lumens narrowing, and others had lost integrity and become fragmented. A layer of young connective tissue with a thickness of 40 ± 14 µm was observed surrounding the macrophage cells, while a lymphocytic infiltrate was present in the connective tissue between the microcapsules (reactivity ranking 2–3). This indicates the onset of microcapsule wall biodegradation.

At the conclusion of a 3-month experiment, the tissues of the omentum containing a functionally active transplant were investigated. In rats with microcapsules after three months (Figure 7D), a less pronounced chronic inflammatory response was observed compared to the two-week and one-month time points. However, the biodegradation of the capsules was in progress, with thickened capsule walls, macrophage layers forming around the capsules, FBGCs on the capsule surfaces, and numerous macrophages containing capsule fragments in their cytoplasm. The microcapsules were deformed, with compromised integrity, and some appeared as eosinophilic homogenous masses. A mature connective tissue layer with a thickness of 45 ± 16 µm surrounded the macrophage cells, while a lymphocytic infiltrate was present in the connective tissue between the implanted microcapsules (reactivity ranking 2). Clusters of islet cells were observed inside a few of the damaged capsules.

**Figure 8:**
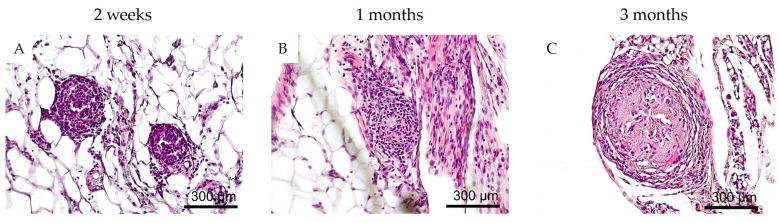
Representative images illustrating the response of surrounding tissues to unencapsulated islet transplantation in rats. (**A**) Total lymphohistiocytic infiltration into the graft at 2 weeks, islet cells cannot be identified; (**B**) fibrosis and moderate lymphocytic infiltration in the area of islet transplantation at 1 month, no transplanted cells are present; (**C**) fibrosis in the graft area at 3 months. Hematoxylin and eosin staining. (Scale bar 300 μm).

Implantation of non-encapsulated rabbit islets (Figure 8) led to a more pronounced inflammatory response at earlier stages (two weeks and one month post-transplantation) compared to the situation with implanted microcapsules. At two weeks, the transplant fragments containing islets were heavily infiltrated by lymphocytes and macrophages, with a few mast cells present. The islet cells could not be identified among the infiltrate. One month post-transplantation, a moderate residual inflammatory response with connective tissue formation was observed, but no islets were found. By three months post-implantation, total fibrosis had developed at the transplant site, with the fibrotic area measuring 153 ± 51 µm, in contrast to the fibrotic tissue surrounding the microcapsules, which measured only 45 ± 16 µm. No islet or acinar tissue was found.

The biocompatibility assessment showed that our novel microcapsules had not provoked a significant inflammatory response, as there were no signs of purulent inflammation or tissue necrosis. This suggests the presence of immunoprotection. The fibrotic overgrowth around the microcapsules was minimal, and no significant differences in fibrosis levels were found across different transplantation time points. The disruption of biostability that did occur was associated with the macrophage response. As a result, the microcapsules lost their integrity approximately one month after transplantation. However, it is important to note that the islet cells they contained were able to maintain their functional activity, highlighting the potential of this approach for prolonging their functionality.

### 3.6. Confirmation of the Maintenance of Functional Activity of Transplanted Encapsulated Islets

**Figure 9:**
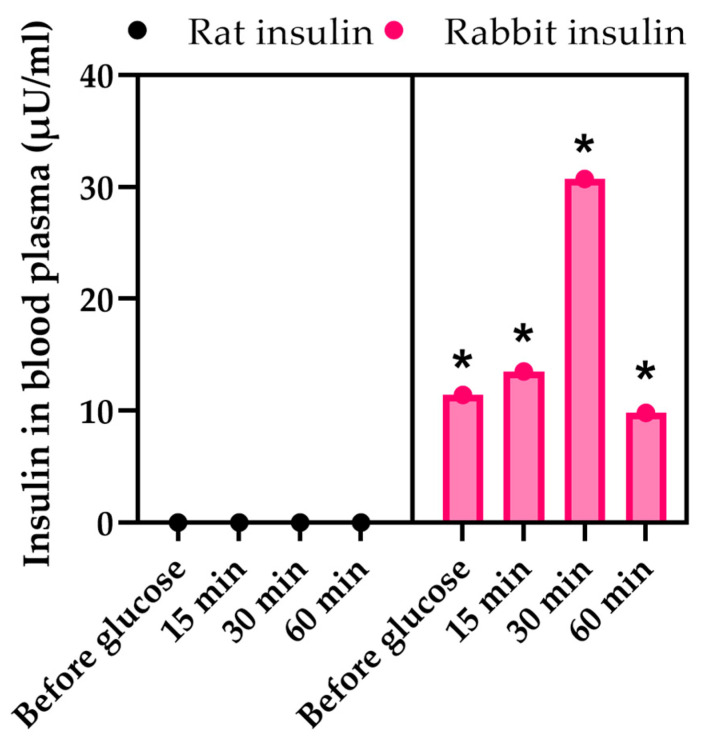
Changes in the concentration of rabbit insulin in the blood of rats with STZ-induced diabetes during GTT following transplantation of encapsulated islets. * *p* < 0.05. Statistical comparisons were made between rabbit insulin and rat insulin levels.

A study of the functional activity of transplanted encapsulated islets was conducted to confirm their ability to continue functioning after capsule degradation. The functional activity of the graft was demonstrated by the results of a glucose tolerance test (GTT) (Figure 9). In rats transplanted with encapsulated rabbit islets, rat insulin was absent from the bloodstream, but rabbit insulin was detected. The level of rabbit insulin increased 30 min after glucose administration, indicating that the encapsulated islets were stimulated by glucose.

**Figure 10:**
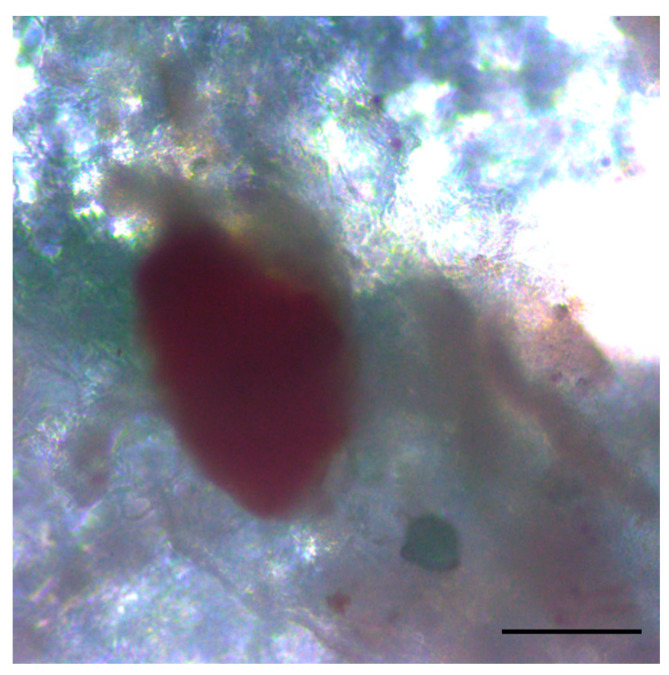
Image of islet in the rat omentum 3 months after transplantation. Dithizone staining. Scale bar 100 µm.

The viability and functional activity of the islets three months after transplantation were confirmed by staining with the specific dye dithizone (Figure 10), which binds to the zinc that participates in insulin granule formation, giving the islets a characteristic red color.

**Figure 11:**
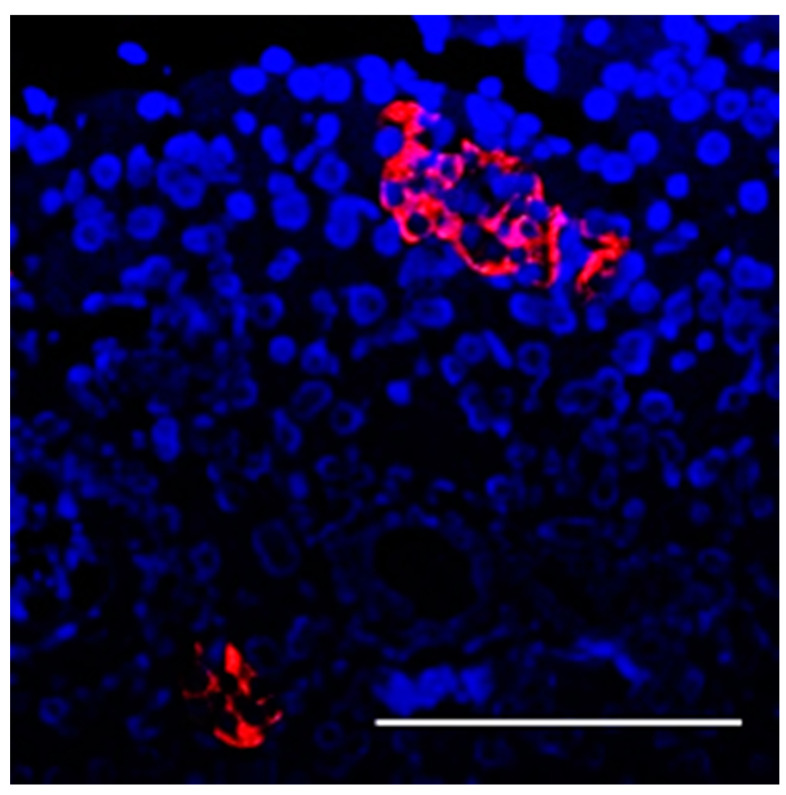
Image of the pancreas of rats 3 months after the transplantation of encapsulated islets. Fluorescence images showing islets labeled with insulin-producing cells (green) and glucagon-producing cells (red) and cell nuclei (blue). (Scale bar 100 μm).

Additionally, the functional activity of transplanted encapsulated islets was demonstrated through a significant reduction in blood glucose levels in rats receiving these transplants. This observation is particularly important given the absence of insulin-producing cells in the pancreas, as confirmed by immunohistochemical analysis (Figure 11). These results underscore the ability of the transplanted cells to compensate for insulin deficiency independently of native pancreatic function. Thus, our study provides compelling evidence for the functional activity of encapsulated islets.

## 4. Discussion

This study is the first time that pancreatic islets encapsulated in alginate microcapsules coated with PMETAC have been tested in vivo. We demonstrated that, after transplantation, the encapsulated islets continued to synthesize insulin, and that the capsule provided immunoprotective properties, extending the functional lifespan of the islets. In our previous work, we had performed a comprehensive in vitro evaluation of these capsules, demonstrating their stability (including osmotic, thermal, and culturing stability) together with their lack of cytotoxicity, all of which indicate their potential for use in islet immunoisolation strategies [13].

The uniqueness of the capsules we studied lies in the fact that the alginate microcapsules coated with PMETAC do not contain copolymers, therefore making them simpler to manufacture and use. Previously, PMETAC has been used by researchers as a copolymer in the production of alginate microcapsules for the encapsulation of C2C12 mouse cells [22,23]. In those studies, it was shown that the permeability of the resulting microcapsules had a threshold value of 70 kDa, potentially making them suitable for islet encapsulation and protection from the immune system described the possible formation of a polyelectrolyte complex between PMETAC and negatively charged proteins (e.g., albumin). Fortunately, the simple step of replacing the medium with a serum-free one after fabrication eliminated any host reactions [23]. Furthermore, PMETAC itself is already used to coat the surfaces of medical devices such as catheters, implants, and surgical instruments to prevent bacterial infections [24]. There is also evidence supporting the biocompatibility of PMETAC in implants. PMETAC allows for the creation of coatings that have been successfully applied to biomaterials [25]. Thus, we hypothesized that PMETAC is a highly promising material for islet encapsulation.

In this study, we demonstrated that islets encapsulated in alginate–PMETAC microcapsules retain their functional activity for a substantial period after transplantation. Although we did not achieve normoglycemia in rats with STZ-induced diabetes after transplantation of the encapsulated islets, we observed a sufficient reduction in blood glucose levels—by 33%. In this study, we demonstrated that islets encapsulated in alginate–PMETAC microcapsules retain their functional activity for a substantial duration following transplantation. Although normoglycemia was not achieved in rats with STZ-induced diabetes following the transplantation of encapsulated islets, a significant reduction in blood glucose levels by 33% was observed. The absence of normoglycemia may be associated with the number of transplanted islets. In this study, 2000 islets were transplanted, which is equivalent to 8000 islets/kg. According to the literature, the requisite number of islets to achieve normoglycemia varies across different studies. For instance, in a study investigating islet allotransplantation in rats, doses ranging from 700 to 1100 islets (approximately 4000 islets/kg) were reported to be necessary to achieve normoglycemia [26], whereas another study indicated that a dose of 10,000 islets/kg was required to attain the same outcome [27]. In xenotransplantation, various doses are also utilized: some studies suggest that normoglycemia can be obtained with doses of 4000 to 7000 islets/kg (from pigs to rats) [16], whereas others report a minimum effective dose of 50,000 islets/kg (from humans to mice) [28]. Nevertheless, some researchers have noted that a 22% reduction in blood glucose is sufficient to demonstrate the functional activity of both syngeneic and allogeneic islet grafts [18]. Indeed, although the primary goal of clinical islet transplantation is to achieve insulin independence in patients with type 1 diabetes, this is difficult to attain in practice. The Edmonton group found that only 10% of patients remained insulin-independent for five years after islet transplantation [29]. Therefore, the current practical goal of islet transplantation therapy is provide blood glucose stabilization, which leads to a significant improvement in disease management and reduces diabetic complications [30]. Thus, maintaining graft functionality and alleviating severe hyperglycemia without immunosuppression remains an important goal.

Furthermore, we demonstrated that the alginate–PMETAC capsules help prolong the functional activity of the islets. While non-encapsulated islets ceased functioning within seven days post-transplantation, approximately 20% of the encapsulated islets maintained functional activity for up to 90 days. Similar studies also report an increase in the functional activity of encapsulated islets compared to non-encapsulated ones [30,31]. The death of non-encapsulated islets cells was likely caused by hyperacute rejection, an effect previously reported by Badet et al. (2002) and Bennet (2000) [32,33]. Hyperacute rejection is a complement-mediated immune response that occurs when there is a discordant immune reaction, causing transplanted xenografts to lose their function rapidly. Preventing contact between the transplanted xenograft and complement can inhibit such hyperacute rejection [30]. Our study used xenogeneic islets as a source of immune attack to confirm the immunoisolating effect of the capsules. Thus, we demonstrated that our novel type of capsule is capable of both prolonging graft function and providing immunoprotective properties.

The evaluation of the anti-fibrotic properties and biostability of the capsules was conducted through a histological analysis of omental tissue after the graft had lost its functional activity. It is currently believed that the main cause of failure in encapsulated cell grafts is through the so-called foreign body response (FBR). FBR is a protective response of the body to almost any implanted devices made from metals, ceramics, or polymers. Even widely used biocompatible materials, including poly(2-hydroxyethyl methacrylate) (PHEMA), polyethylene glycol (PEG), and alginate hydrogels, can trigger a significant FBR [34]. An FBR is a complex process involving signaling cascades and cellular responses, typically described in four stages: protein adsorption, acute inflammation, chronic inflammation, and fibrosis. The fibrotic capsule that forms 3–4 weeks post-implantation isolates the graft from the surrounding tissue, impairing the diffusion of oxygen, nutrients, and metabolic waste, ultimately leading to cell death. Various approaches exist to reduce the FBR to grafts, one of which is the application of new polymer coatings [35]. For instance, zwitterionic polymers composed of sulfobetaine methacrylate (SB) and 2-aminoethyl methacrylate (AE) were investigated for their ability to mitigate the foreign body response in vivo when used to modify polydimethylsiloxane (PDMS) samples. The implants were subcutaneously transplanted into rats. A significant reduction in capsule thickness of 52.6% was observed in PDMS samples treated with zwitterionic compounds, with fibrous capsule thickness measured at 142 ± 23 μm, compared to 270 ± 49 μm for untreated samples [36]. In our study, the fibrotic overgrowth around the new xenogeneic islet-containing capsules (45 ± 16 µm) was 79% less pronounced than around non-encapsulated xenogeneic islets (153 ± 51 µm). This indicates that the new PMETAC-coated capsule can reduce the extent of FBR.

However, it was found that this type of capsule biodegrades due to the macrophage response, as evidenced by the large number of multinucleated giant foreign body cells surrounding the swollen capsules and the fragmented capsule material lying freely in the tissue. However, although the capsules had lost their integrity by one month after transplantation, in some cases, the islets they had contained were able to maintain their functional activity, as demonstrated by the reduced blood glucose levels in rats with capsule transplants compared to untreated animals. Further support for this conclusion was provided by the immunohistochemical (IHC) and ELISA results and the presence of islets in the omentum three months after transplantation, as was demonstrated by dithizone staining. However, this effect was observed in only 20% of the animals. In the others, the loss of islet function was likely due to capsule biostability failure. While there have been studies that have monitored glucose levels and used fibrosis assessment for longer periods [37], we believe that PMETAC remains a promising polymer for further capsule refinement.

Thus, our novel capsule with its unique combination of alginate and PMETAC polymers does not cause significant fibrosis yet supports the survival of viable islets. In the future, the capsule may be further improved and stabilized to extend its integrity and to reduce the macrophage response.

## 5. Conclusions

In this study, we successfully demonstrated that alginate–PMETAC microcapsules significantly prolong the functional activity of pancreatic islets transplanted into rats with STZ-induced diabetes. Unlike non-encapsulated ones, the encapsulated islets maintained insulin secretion and thus reduced hyperglycemia, highlighting the immunoprotective and biocompatible properties of the capsule material. The minimal fibrotic overgrowth and sustained graft function indicate that PMETAC is a promising candidate for further development in cell encapsulation technology. The current challenges such as capsule biodegradation indicate the need for future improvements in microcapsule design in order to enhance their clinical application in diabetes and other disorders requiring cell-based therapies, so future work will focus on increasing the biostability of the capsules.

## Data Availability

Data are available on request from the corresponding author.
